# Guanidinium chloro­chromate

**DOI:** 10.1107/S1600536810002710

**Published:** 2010-01-27

**Authors:** Hoong-Kun Fun, Jia Hao Goh, Arnab Kar, Shyamaprosad Goswami

**Affiliations:** a X-ray Crystallography Unit, School of Physics, Universiti Sains Malaysia, 11800 USM, Penang, Malaysia; b Department of Chemistry, Bengal Engineering and Science University, Shibpur, Howrah 711 103, India

## Abstract

In the title compound, guanidinium chloridotrioxidochrom­ate(VI), (CH_6_N_3_)[CrClO_3_], both the cation and anion are generated by crystallographic mirror symmetry, with one O and one N atom and the Cr, Cl and C atoms lying on the mirror plane. The bond lengths in the guanidinium cation are inter­mediate between normal C—N and C=N bond lengths, indicating significant delocalization in this species. In the crystal structure, inter­molecular N—H⋯Cl inter­actions generate *R*_2_^1^(6) ring motifs. These ring motifs are further inter­connected by inter­molecular N—H⋯O hydrogen bonds into infinite chains along [010].

## Related literature

For background to chlorido­chromates in organic synthesis, see: Ghammaamy & Maza­reey (2005 ▶). For bond-length data, see: Allen *et al.* (1987 ▶). For graph-set descriptions of hydrogen-bond motifs, see: Bernstein *et al.* (1995 ▶). For related structures, see: Al-Dajani *et al.* (2009 ▶); Lorenzo Luis *et al.* (1996 ▶). For the stability of the temperature controller used for the data collection, see: Cosier & Glazer (1986 ▶).
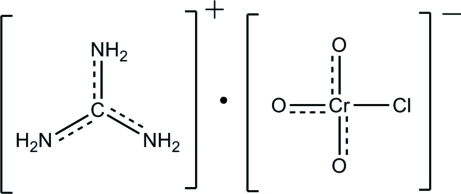


## Experimental

### 

#### Crystal data

(CH_6_N_3_)[CrClO_3_]
*M* *_r_* = 195.54
Orthorhombic, 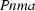

*a* = 5.9708 (2) Å
*b* = 7.5302 (2) Å
*c* = 14.7085 (4) Å
*V* = 661.31 (3) Å^3^
*Z* = 4
Mo *K*α radiation
μ = 2.07 mm^−1^
*T* = 100 K
0.85 × 0.20 × 0.07 mm

#### Data collection

Bruker SMART APEXII CCD area-detector diffractometer
Absorption correction: multi-scan (*SADABS*; Bruker, 2009 ▶) *T*_min_ = 0.273, *T*_max_ = 0.875
11570 measured reflections
1843 independent reflections
1727 reflections with *I* > 2σ(*I*)
*R*_int_ = 0.027

#### Refinement

*R*[*F* ^2^ > 2σ(*F* ^2^)] = 0.020
*wR*(*F* ^2^) = 0.054
*S* = 1.10
1843 reflections
61 parameters
All H-atom parameters refined
Δρ_max_ = 0.55 e Å^−3^
Δρ_min_ = −0.41 e Å^−3^

### 

Data collection: *APEX2* (Bruker, 2009 ▶); cell refinement: *SAINT* (Bruker, 2009 ▶); data reduction: *SAINT*; program(s) used to solve structure: *SHELXTL* (Sheldrick, 2008 ▶); program(s) used to refine structure: *SHELXTL*; molecular graphics: *SHELXTL*; software used to prepare material for publication: *SHELXTL* and *PLATON* (Spek, 2009 ▶).

## Supplementary Material

Crystal structure: contains datablocks global, I. DOI: 10.1107/S1600536810002710/hb5314sup1.cif

Structure factors: contains datablocks I. DOI: 10.1107/S1600536810002710/hb5314Isup2.hkl

Additional supplementary materials:  crystallographic information; 3D view; checkCIF report

## Figures and Tables

**Table 1:** Selected bond lengths (Å)

Cr1—O2	1.6101 (6)
Cr1—O2	1.6183 (8)
Cr1—Cl1	2.2099 (3)

**Table 2:** Hydrogen-bond geometry (Å, °)

*D*—H⋯*A*	*D*—H	H⋯*A*	*D*⋯*A*	*D*—H⋯*A*
N1—H1*N*1⋯O2^ii^	0.806 (14)	2.211 (13)	2.9984 (9)	165.7 (12)
N2—H1*N*2⋯O1^iii^	0.817 (14)	2.192 (14)	2.9702 (8)	159.4 (13)
N2—H2*N*2⋯Cl1	0.812 (13)	2.753 (13)	3.5075 (8)	155.5 (13)

Symmetry codes: (ii) 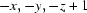; (iii) 
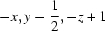
.

**Fig. 1.**
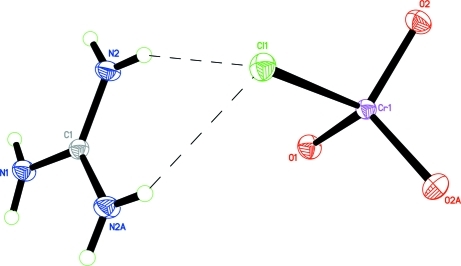
The molecular structure of (I), showing 50% probability displacement ellipsoids for non-H atoms. The suffix A corresponds to the symmetry code [x, -y+1/2, z]. Intermolecular N—H···Cl hydrogen bonds are shown as dashed lines.

**Fig. 2.**
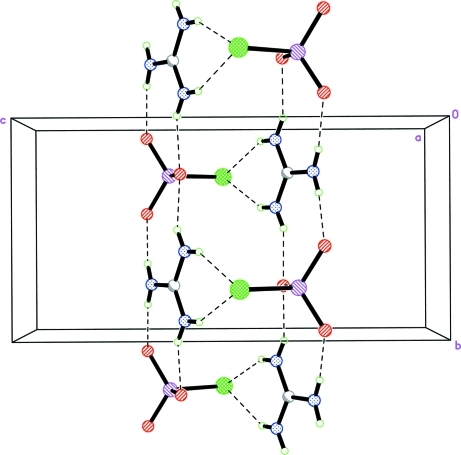
The crystal structure of (I), viewed along the a axis, showing R12(6) ring motifs being interconnected into one-dimensional chain along the b axis. Intermolecular hydrogen bonds are shown as dashed lines.

## supplementary crystallographic information

### Comment

Many methods and oxidizing agents for organic synthesis are known but development and modification of known reagents have only been studied in recent years, e.g. tributylammonium chlorochromate (Ghammaamy & Mazareey, 2005).

The asymmetric unit of the title compound comprises of a guanidinium cation and a chlorochromate anion (Fig. 1). Both of the cation and anion lie on a crystallographic mirror plane and contain one-half molecule [symmetry code of atoms labelled with suffix A: x, -y+1/2, z]. The coordination geometry formed by three O atoms and a Cl atom around the Cr atom is distorted tetrahedral, as indicated by the O1—Cr1—Cl1 and O2—Cr1—O2A angles of 106.21 (3) and 112.06 (4)° respectively. The C1–N1 and C1–N2 bond lengths in the propeller-shaped guanidinium cation are almost equal [1.3310 (14) and 1.3289 (8) Å respectively], indicating that the usual model of electron dislocalization in this moiety (Allen *et al.*,  1987). The bond lengths and angles are comparable to closely related guanidinium (Al-Dajani *et al.*, 2009) and chlorochromate (Lorenzo Luis *et al.*, 1996) structures.

In the crystal structure (Fig. 2), all guanidinium-H atoms participate in intermolecular hydrogen bonds. Intermolecular N2—H2N2···Cl1 interactions (Table 1) form bifurcated acceptor hydrogen bonds which generate *R*^1^_2_(6) ring motifs (Bernstein *et al.*, 1995). These ring motifs are further interconnected into one-dimensional infinite chain along the [010] direction by intermolecular N1—H1N1···O2 and N2—H1N2···O1 hydrogen bonds (Table 1).

### Experimental

The new oxidizing reagent, guanidinium chlorochromate (GCC) was prepared by treatment of equivalent amounts of guanidinium hydrochloride and chromium trioxide in water at 273 K by stirring with a glass rod with instantaneous formation of yellow blocks of (I).

### Refinement

All the H atoms were located from difference Fourier map and allowed to refine freely [Range of N—H = 0.805 (14) – 0.818 (14) Å].

### Crystal data

**Table:**

(CH_6_N_3_)[CrClO_3_]	*F*(000) = 392
*M**_r_* = 195.54	*D*_x_ = 1.964 Mg m^−^^3^
Orthorhombic, *P**n**m**a*	Mo *K*α radiation, λ = 0.71073 Å
Hall symbol: -P 2ac 2n	Cell parameters from 7278 reflections
*a* = 5.9708 (2) Å	θ = 2.8–40.1°
*b* = 7.5302 (2) Å	µ = 2.07 mm^−^^1^
*c* = 14.7085 (4) Å	*T* = 100 K
*V* = 661.31 (3) Å^3^	Plate, yellow
*Z* = 4	0.85 × 0.20 × 0.07 mm

### Data collection

**Table:**

Bruker SMART APEXII CCD area-detector diffractometer	1843 independent reflections
Radiation source: fine-focus sealed tube	1727 reflections with *I* > 2σ(*I*)
graphite	*R*_int_ = 0.027
φ and ω scans	θ_max_ = 37.5°, θ_min_ = 2.8°
Absorption correction: multi-scan (*SADABS*; Bruker, 2009)	*h* = −8→10
*T*_min_ = 0.273, *T*_max_ = 0.875	*k* = −12→12
11570 measured reflections	*l* = −21→25

### Refinement

**Table:**

Refinement on *F*^2^	Primary atom site location: structure-invariant direct methods
Least-squares matrix: full	Secondary atom site location: difference Fourier map
*R*[*F*^2^ > 2σ(*F*^2^)] = 0.020	Hydrogen site location: inferred from neighbouring sites
*wR*(*F*^2^) = 0.054	All H-atom parameters refined
*S* = 1.10	*w* = 1/[σ^2^(*F*_o_^2^) + (0.0252*P*)^2^ + 0.1555*P*] where *P* = (*F*_o_^2^ + 2*F*_c_^2^)/3
1843 reflections	(Δ/σ)_max_ < 0.001
61 parameters	Δρ_max_ = 0.55 e Å^−^^3^
0 restraints	Δρ_min_ = −0.41 e Å^−^^3^

### Special details

**Table:**

Experimental. The crystal was placed in the cold stream of an Oxford Cyrosystems Cobra open-flow nitrogen cryostat (Cosier & Glazer, 1986) operating at 100.0 (1)K.
Geometry. All esds (except the esd in the dihedral angle between two l.s. planes) are estimated using the full covariance matrix. The cell esds are taken into account individually in the estimation of esds in distances, angles and torsion angles; correlations between esds in cell parameters are only used when they are defined by crystal symmetry. An approximate (isotropic) treatment of cell esds is used for estimating esds involving l.s. planes.
Refinement. Refinement of F^2^ against ALL reflections. The weighted R-factor wR and goodness of fit S are based on F^2^, conventional R-factors R are based on F, with F set to zero for negative F^2^. The threshold expression of F^2^ > 2sigma(F^2^) is used only for calculating R-factors(gt) etc. and is not relevant to the choice of reflections for refinement. R-factors based on F^2^ are statistically about twice as large as those based on F, and R- factors based on ALL data will be even larger.

### Fractional atomic coordinates and isotropic or equivalent isotropic displacement parameters (Å^2^)

**Table:**

	*x*	*y*	*z*	*U*_iso_*/*U*_eq_	
Cr1	0.38355 (3)	0.2500	0.646530 (11)	0.00989 (5)	
Cl1	0.57743 (4)	0.2500	0.518544 (17)	0.01422 (6)	
O1	0.12221 (13)	0.2500	0.61735 (6)	0.01476 (14)	
O2	0.44850 (10)	0.07267 (8)	0.70172 (4)	0.01594 (11)	
N1	−0.15734 (17)	0.2500	0.31553 (7)	0.01472 (16)	
N2	0.11145 (12)	0.09721 (9)	0.39621 (5)	0.01507 (12)	
C1	0.02125 (18)	0.2500	0.36969 (7)	0.01107 (15)	
H1N1	−0.218 (2)	0.1568 (19)	0.3047 (8)	0.024 (3)*	
H1N2	0.060 (2)	0.0026 (19)	0.3789 (9)	0.024 (3)*	
H2N2	0.221 (2)	0.0981 (19)	0.4289 (9)	0.027 (3)*	

### Atomic displacement parameters (Å^2^)

**Table:**

	*U*^11^	*U*^22^	*U*^33^	*U*^12^	*U*^13^	*U*^23^
Cr1	0.01033 (8)	0.00893 (7)	0.01042 (7)	0.000	−0.00080 (5)	0.000
Cl1	0.01339 (11)	0.01634 (11)	0.01294 (10)	0.000	0.00166 (8)	0.000
O1	0.0114 (3)	0.0166 (3)	0.0163 (3)	0.000	−0.0014 (3)	0.000
O2	0.0184 (3)	0.0132 (2)	0.0162 (2)	0.00135 (19)	−0.0015 (2)	0.00329 (18)
N1	0.0154 (4)	0.0121 (3)	0.0166 (4)	0.000	−0.0047 (3)	0.000
N2	0.0169 (3)	0.0099 (2)	0.0184 (3)	0.0013 (2)	−0.0047 (2)	0.0001 (2)
C1	0.0120 (4)	0.0106 (3)	0.0106 (4)	0.000	0.0014 (3)	0.000

### Geometric parameters (Å, °)

**Table:**

Cr1—O2	1.6101 (6)	N1—H1N1	0.805 (14)
Cr1—O2^i^	1.6102 (6)	N2—C1	1.3289 (8)
Cr1—O1	1.6183 (8)	N2—H1N2	0.818 (14)
Cr1—Cl1	2.2099 (3)	N2—H2N2	0.811 (14)
N1—C1	1.3310 (14)	C1—N2^i^	1.3289 (8)
			
O2—Cr1—O2^i^	112.06 (4)	C1—N2—H1N2	120.7 (9)
O2—Cr1—O1	111.47 (3)	C1—N2—H2N2	119.5 (10)
O2^i^—Cr1—O1	111.47 (3)	H1N2—N2—H2N2	119.8 (14)
O2—Cr1—Cl1	107.66 (2)	N2^i^—C1—N2	119.94 (10)
O2^i^—Cr1—Cl1	107.66 (2)	N2^i^—C1—N1	120.02 (5)
O1—Cr1—Cl1	106.21 (3)	N2—C1—N1	120.02 (5)
C1—N1—H1N1	118.5 (9)		

Symmetry codes: (i) *x*, −*y*+1/2, *z*.

### Hydrogen-bond geometry (Å, °)

**Table:**

*D*—H···*A*	*D*—H	H···*A*	*D*···*A*	*D*—H···*A*
N1—H1N1···O2^ii^	0.806 (14)	2.211 (13)	2.9984 (9)	165.7 (12)
N2—H1N2···O1^iii^	0.817 (14)	2.192 (14)	2.9702 (8)	159.4 (13)
N2—H2N2···Cl1	0.812 (13)	2.753 (13)	3.5075 (8)	155.5 (13)

Symmetry codes: (ii) −*x*, −*y*, −*z*+1; (iii) −*x*, *y*−1/2, −*z*+1.
